# The LoVe score: Development and validation of a bedside clinical index to identify patients at risk for prolonged invasive mechanical ventilation

**DOI:** 10.1177/17511437261479649

**Published:** 2026-08-28

**Authors:** Paul A. Hilders, Lada Lijović, Martijn Otten, Laurens A. Biesheuvel, Ameet R. Jagesar, Olof Sigling, Paul W. G. Elbers

**Affiliations:** 1 Department of Intensive Care Medicine, Center for Critical Care Computational Intelligence, Amsterdam Medical Data Science (AMDS), Amsterdam Cardiovascular Science (ACS), Amsterdam Public Health (APH), Amsterdam Institute for Immunology and Infectious Diseases (AII), Amsterdam UMC, Vrije Universiteit Amsterdam, The Netherlands; 2 Computer Science Department, Vrije Universiteit Amsterdam, The Netherlands; 3 Department of Anesthesiology, Intensive Care and Pain Management, University Hospital Center Sestre Milosrdnice, Zagreb, Croatia

**Keywords:** mechanical ventilation, retrospective studies, machine learning, index scores, duration of therapy

## Abstract

**Background:**

Early management decisions after intubation, such as humidification strategy or initiation of prevention bundles for ventilator-associated pneumonia, often depend on the expected duration of invasive mechanical ventilation (IMV). However, no simple, standardised method exists to support such estimates at the bedside. We aimed to develop and validate the Length of Ventilation (LoVe) index, a score to predict prolonged IMV beyond 48 h using peri-intubation data.

**Methods:**

We conducted a retrospective cohort study using two large, publicly available ICU databases: AmsterdamUMCdb for model development and internal validation, and MIMIC-IV for external validation. The primary outcome was prolonged IMV, defined as support beyond 48 h or death within 48 h. Two LoVe variants were derived: a ventilator-inclusive version incorporating ventilator settings, and a ventilator-agnostic version without ventilator settings. Predictors identified via logistic regression were discretised into clinically interpretable ranges and summed into an additive score. Clinical utility was assessed using decision curve analysis.

**Results:**

In the cardiac surgery and non-cardiac surgery cohorts, the ventilator-inclusive LoVe index achieved AUROC scores of 0.82 and 0.75, respectively. The ventilator-agnostic version reached 0.72 in the non-cardiac surgery cohort. Validation in MIMIC-IV yielded 0.71 and 0.68 for the ventilator-inclusive version, and 0.71 and 0.65 for the ventilator-agnostic index. Decision curve analysis demonstrated that both LoVe indices provided meaningful clinical utility.

**Conclusions:**

The LoVe index provides a simple, interpretable bedside tool to estimate the likelihood of prolonged IMV early after intubation, showing consistent external validity and potential to support early ICU decision-making.

## Introduction

Prolonged invasive mechanical ventilation (IMV) is common among critically ill ICU patients and is linked to worse outcomes, including higher mortality, longer ICU stays, and increased costs.^1–4^ Patients requiring extended support are also at elevated risk of complications such as ventilator-associated pneumonia, limb muscle atrophy, airway obstruction, and diaphragm dysfunction.^5–7^ Early estimation of ventilation duration at initiation can guide key clinical decisions, from resource allocation to setting expectations with patients and families.

From a resource-allocation standpoint, humidification strategy is an early management decision guided by the expected duration of mechanical ventilation. Guidelines recommend heat and moisture exchangers (HMEs) for short-term support and switching to heated humidifiers (HHs) when ventilation is anticipated to exceed a defined interval, such as 96 h per the American Association for Respiratory Care (AARC) guidelines.^8^ Despite these recommendations, clinical studies on the comparative effectiveness of HH versus HME have adopted markedly different approaches to patient selection and intervention allocation. Whereas some trials assign patients to HH or HME directly after initiation of ventilation, irrespective of likely duration,^9^ other studies include only patients who have already remained ventilated for a fixed period (e.g. 48 h).^10^ A third group of studies allocates HH versus HME based on the clinician’s subjective expectation of ventilation duration, at the time of ventilation initiation.^8,11^ These divergent strategies likely contribute to substantial international variability in humidification practices^12^ and underscore the absence of a standardised, objective method to estimate ventilation duration at initiation.

While humidification strategy is only one of several management decisions influenced by expected ventilation duration, it exemplifies how the absence of a reliable prediction method can lead to variability in both clinical practice and research design. Beyond humidification, early decisions such as starting ventilator-associated pneumonia (VAP) prevention bundles, lung- and diaphragm-protective strategies including early prone positioning, analgosedation protocols, delirium prevention bundles, early involvement of respiratory therapy, starting selective decontamination of the digestive tract, and initiation of enteral nutrition, are frequently contingent on anticipated ventilation duration. An accurate, reproducible tool available at the time of intubation could standardise intervention allocation, support guideline adherence, and improve comparability across studies.

Although several models have been developed to predict the duration of mechanical ventilation, most of these studies lack practical utility. For instance, prognostic scores such as the ANZ-PreVent^13^ and Modified Hessels et al.^14^ indices focus on perioperative risk factors to predict ventilation beyond 24 h, in cardiac surgery patients, but these models are not generalisable to the broader ICU population and address a relatively short time horizon. Some models estimate duration in days rather than classifying prolonged IMV,^15^ while others rely on data after ARDS onset or within the first 48–72 h, limiting utility at intubation.^16,17^ Still others target longer horizons, predicting prolonged IMV as >7 days or >14 days,^18,19^ and are less applicable to early ICU decision-making. Collectively, these studies show that prognostic modelling of ventilation duration is feasible but also reveal the lack of a simple, interpretable tool to predict prolonged IMV beyond 48 h using only data available at or just before intubation.

In this study, we therefore develop and validate the Length of invasive mechanical Ventilation (LoVe) score, a simple, interpretable bedside index for the prediction of prolonged mechanical ventilation beyond 48 h, using routinely available clinical data from the peri-intubation period.

## Methods

This study is reported following the Transparent Reporting of a multivariable prediction model for Individual Prognosis Or Diagnosis (TRIPOD) guidelines.^20^ The derivation and internal analyses were performed using AmsterdamUMCdb, a publicly accessible database consisting of de-identified health data from 23,106 admissions to the ICU of Amsterdam University Medical Center in the Netherlands between 2003 and 2016.^21^ The dataset used for external validation was extracted from the MIMIC-IV database, which contains de-identified medical data for 73,181 admissions to an ICU at the Beth Israel Deaconess Medical Center between 2008 and 2019.^22^

## Study population

All adult, critically ill patients who received invasive mechanical ventilation (IMV) during their admission were deemed eligible for inclusion. Patients were only excluded if the type of ventilatory support (e.g. invasive vs non-invasive) could not be reliably determined throughout their ICU stay, ensuring that only confirmed IMV sessions entered the analyses.

## Feature sets and data aggregation

Candidate predictors were compiled from a broad set of routinely collected peri-intubation variables judged potentially informative on the basis of clinical relevance, and subsequently filtered by availability across both datasets. The final feature set included four demographics: admission age, weight, height, and gender; eight vital signs: heart rate, systolic blood pressure, mean blood pressure, diastolic blood pressure, respiratory rate, temperature, oxygen saturation (SpO_2_), and glucose; 21 laboratory values: lactate, pH, partial pressure of oxygen (PaO_2_), partial pressure of carbon dioxide (PaCO_2_), white blood cell count (WBC), haematocrit, haemoglobin, platelets, bicarbonate, blood urea nitrogen (BUN), calcium, chloride, creatinine, glucose, sodium, potassium, albumin, international normalised ratio (INR), partial thromboplastin time (PTT), aspartate transaminase (AST), and alanine transaminase (ALT); and three mechanical ventilator settings: fraction of inspired oxygen (FiO_2_), positive end-expiratory pressure (PEEP), and tidal volume.

Vital signs were aggregated by taking the mean across a prespecified measurement window. Other numerical values, such as the laboratory values and ventilator settings, were aggregated by taking the last documented value within the window, reflecting real-world availability.

We also evaluated the potential inclusion of dynamic respiratory system compliance as a predictor, given its clinical relevance for assessing respiratory mechanics. In preliminary analyses, the approximated compliance demonstrated predictive performance comparable to the ventilator setting variables. However, as it added complexity to the feature set without yielding meaningful performance gains, we did not retain it in the final model. The estimation procedure and the assessment leading to its exclusion are described in Appendix A1.

## Outcome definitions

The primary focus of this study is targeted on the prediction of prolonged IMV, defined a priori as IMV duration exceeding 48 h from the documented start of invasive respiratory support. We use the term prolonged IMV rather than prolonged mechanical ventilation (PMV), deliberately, as PMV is an established concept denoting ventilation over substantially longer horizons.^23^ In contrast, the 48 h threshold reflects the early management decisions the index is intended to support, such as humidification strategy or the initiation of VAP prevention bundles, which require a considerably shorter forecasting window.

In order to address the competing risk of early death, where patients who die before 48 h can never meet the prolonged IMV criterion, we incorporate two variants of the outcome definition (1) Composite outcome: Mechanical ventilation duration > 48 h or death before 48 h; and (2) Exclusion outcome: Mechanical ventilation duration > 48 h, excluding patients who died before 48 h.

This competing risk framework was chosen to ensure that early mortality did not bias model performance or interpretation. Through the exclusion outcome, we target predictors of prolonged IMV specifically, while the composite outcome is better aligned with how the model would be used in clinical practice. Unless explicitly stated otherwise, all reported results are based on the composite outcome definition.

## Dataset configurations

To evaluate practical deployment scenarios, we assessed the index score derivation and performance under various configurations.

### Measurement and prediction windows

First, we consider three different prespecified peri-intubation measurement windows, which determine what measurements to use in the derivation of the score and at what time a prediction can be provided. (1) Early prediction window: data from 4 h preceding intubation until 1 h after intubation; (2) short-term post-intubation prediction window: Data from the time of intubation until 1 h after; (3) long-term post-intubation prediction window: Data from the time of intubation until 6 h after.

For each configuration, predictors were extracted and aggregated within the specified window, and the prediction timepoint was anchored at the end of the window, when the score would be available in practice. Outcome assessment was defined independently of the measurement window to avoid information leakage. By comparing different timeframes, we gain better insight into how the measurement and prediction windows affect the performance and robustness of the derived index score.

### Separation of cardiac surgery patients

As cardiac surgery patients at the derivation site were expected to exhibit a markedly higher proportion of short-term duration IMV, we decided a priori to stratify reporting to avoid misleading aggregate performance metrics. Although we therefore report the results for cardiac surgery and non-cardiac surgery patients separately within this cohort, the same index and thresholds were applied for both subgroups.

## Missing data, outlier detection, and value imputation

After data extraction, we first excluded ventilation sessions with more than 30% missing data, reflecting a pragmatic balance between retaining a representative, adequately sized cohort and excluding sessions too sparsely documented to support reliable downstream use. Then, we also removed features with more than 25% missing values and features that exhibited strong collinearity (i.e. Pearson’s correlation coefficient > 0.6) with other variables. We performed outlier detection and removal based on handpicked value thresholds, determined by clinical expertise. For the remainder of the incomplete features, we used median imputation to fill in the missing values.

## Model development and score derivation

Model derivation proceeded in four steps, repeated for each configuration and index variant. First, we trained a logistic regression model using all candidate predictors to estimate associations with prolonged IMV. Second, we combined insights from Shapley Additive exPlanations (SHAP) values with the standardised model coefficients to select a subset of clinically plausible predictors. Third, we discretised the continuous predictors into clinically interpretable ranges based on an empirical assessment of the distributional differences in the prolonged IMV and non-prolonged IMV groups, as described in Appendix A2. Each derived predictor range was then assigned a point value between 0 and 3, with higher values indicating a higher likelihood of prolonged IMV. Finally, we trained a new logistic regression model using only the resulting additive index score as a feature, in order to evaluate predictive performance and facilitate calibration.

## Internal/external validation and evaluation metrics

The dataset derived from AmsterdamUMCdb was split by patient into training and test sets using a random 80%–20% split. The final score was then applied to the MIMIC-IV external validation cohort. The predictive performance of the models was assessed on the held-out test dataset using sensitivity, specificity, and the Area Under the Receiver Operating Characteristic (AUROC) curve. Class imbalance was addressed with oversampling and 95% confidence intervals (CIs) were approximated using bootstrapping.

In addition, we performed a decision curve analysis (DCA) to evaluate clinical utility of our index score across a clinically plausible range of threshold probabilities.^24^

## Data availability

In this study, we used publicly available data from the AmsterdamUMCdb and MIMIC-IV databases, which are available to researchers upon completion of the CITI Data or Specimens Only Research training. The source code used to produce the results and analyses presented in this manuscript is available upon reasonable request to the authors.

## Results

**Figure 1.**
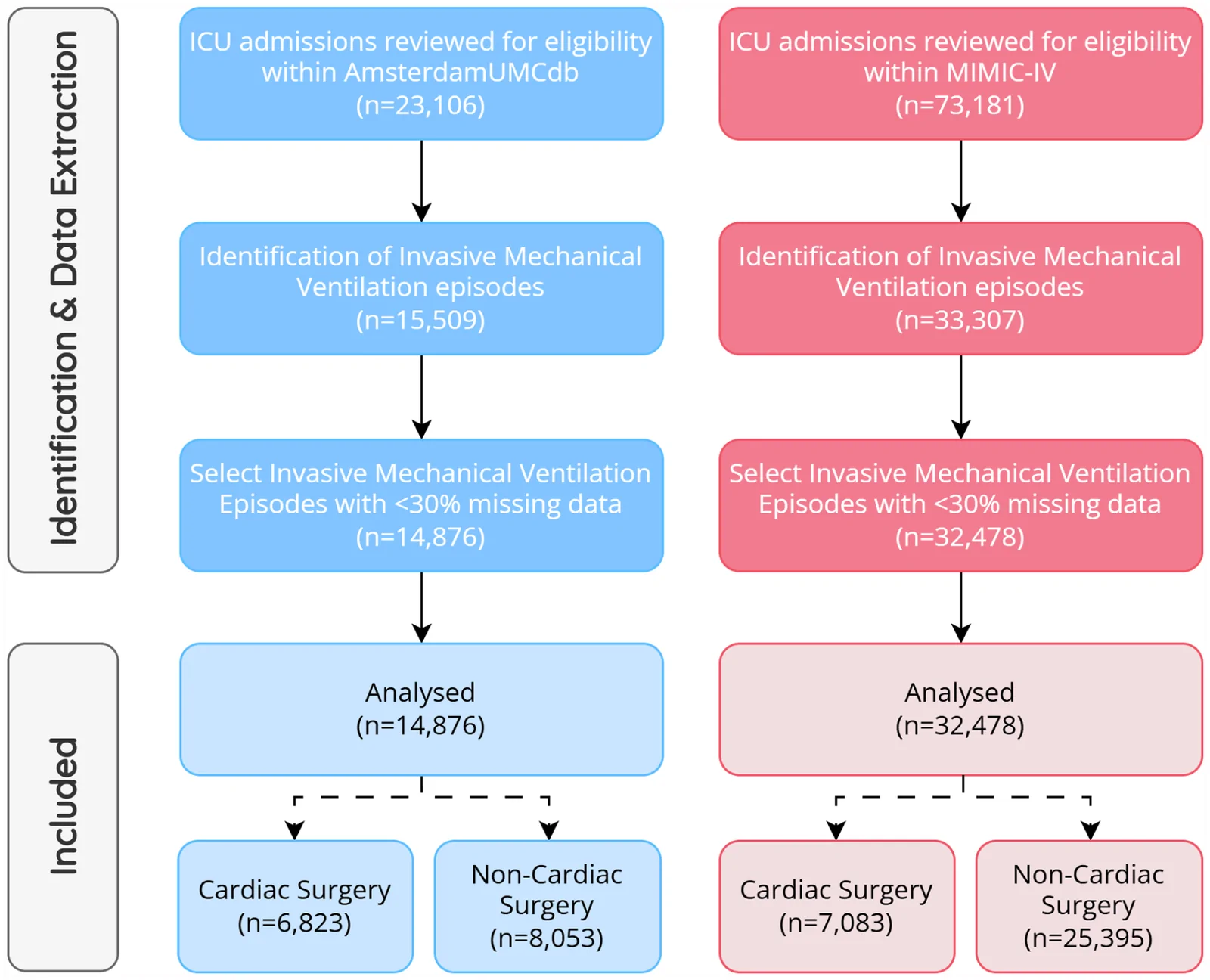
Inclusion flowchart for the AmsterdamUMCdb and MIMIC-IV datasets, using the early prediction window.

Following our specified inclusion and exclusion criteria, we identified 15,509 and 33,307 invasive mechanical ventilation episodes for the AmsterdamUMCdb and MIMIC-IV databases, respectively. From these episodes, we selected 14,876 and 32,478 IMV sessions which did not exceed our specified data missingness threshold of 30%. In the AmsterdamUMCdb cohort, roughly 45.6% of these, or 6823 episodes, corresponded to cardiac surgery patients, while in the MIMIC-IV cohort, 21.8% or 7083 episodes were identified as cardiac surgery cases. A visualisation of the inclusion flowchart for the early prediction window is displayed in Figure 1.

Baseline characteristics for the AmsterdamUMCdb and MIMIC-IV cohorts are presented in Table 1. Given the multiple dataset configurations and peri-intubation timeframes considered in this study, we report the characteristics of the cohorts at the stage of IMV episode identification, prior to the application of configuration-specific inclusion criteria (see Figure 1). This approach ensures a consistent overview of baseline demographics and clinical features across cohorts. However, the final analytic cohorts used for model development and validation may differ slightly in composition, particularly due to subsequent exclusions based on timeframe-specific data availability or missingness.

**Table 1.** Baseline cohort characteristics.

Characteristic	AUMCdb CS	AUMCdb non-CS	MIMIC-IV CS	MIMIC-IV non-CS
*n*	6943	8566	7140	26,167
Age (years), mean (SD)	67.5 (10.9)	60.9 (16.6)	68.2 (12.2)	63.9 (16.8)
Gender (Male), *n* (%)	5072 (73.1)	5625 (65.7)	4841 (67.8)	15,017 (57.4)
Weight (kg), mean (SD)	82.6 (13.9)	79.2 (14.4)	84.5 (20.3)	82.5 (26.4)
Height (cm), mean (SD)	174 (9)	176 (9)	170 (10)	169 (11)
IMV Duration (h), mean (SD)	26.9 (111.0)	119.7 (216.6)	22.7 (39.5)	38.9 (59.3)
ICU LOS (days), mean (SD)	2.6 (7.3)	9.1 (13.5)	8.6 (13.2)	11.3 (12.4)

CS: cardiac surgery; SD: standard deviation; IMV: invasive mechanical ventilation; ICU: intensive care unit; LOS: length of stay; AUMCdb: AmsterdamUMCdb.

Since degrees of missingness varied across peri-intubation timeframes, IMV episode exclusion was applied separately for each configuration. In the AmsterdamUMCdb cohort, 633, 727, and 4052 IMV episodes were excluded for the early (−4 h to +1 h), short-term (0 h to +1 h), and long-term (0 h to +6 h) prediction windows, respectively. In the MIMIC-IV cohort, 829 episodes were excluded for the early window and 213 for the long-term window. The short-term window proved overly restrictive in this cohort, requiring the exclusion of several additional features. Since MIMIC-IV was used solely for external validation, we omitted the short-term window from these analyses.

Within the candidate feature set, AST, ALT, BUN, chloride, and lactate were removed due to exceeding a 25% missingness threshold. In addition, systolic and diastolic blood pressure, haematocrit, and glucose (lab) values were excluded due to high collinearity with other features. The final feature set used for model development and validation comprised 27 variables.

## Full model prediction performance

We first evaluated the predictive performance of the full logistic regression model using all candidate features in the AmsterdamUMCdb non-cardiac surgery cohort, focussing on the early prediction window (−4 h to +1 h). For the composite outcome, the model achieved an AUROC of 0.776 (95% CI: 0.771–0.780), sensitivity of 0.659 (0.644–0.674), and specificity of 0.763 (0.749–0.777). Results for the exclusion outcome were comparable, with an AUROC of 0.758 (0.753–0.762), sensitivity of 0.656 (0.641–0.670), and specificity of 0.742 (0.728–0.757). As performance between the two outcome definitions was highly consistent in subsequent analyses, we focus on the composite outcome for the remainder of the results. In the cardiac surgery cohort, performance of the full model was higher, with an AUROC of 0.842 (0.820–0.860), sensitivity of 0.665 (0.622–0.720), and specificity of 0.869 (0.853–0.885).

## Index score derivation

**Figure 2.**
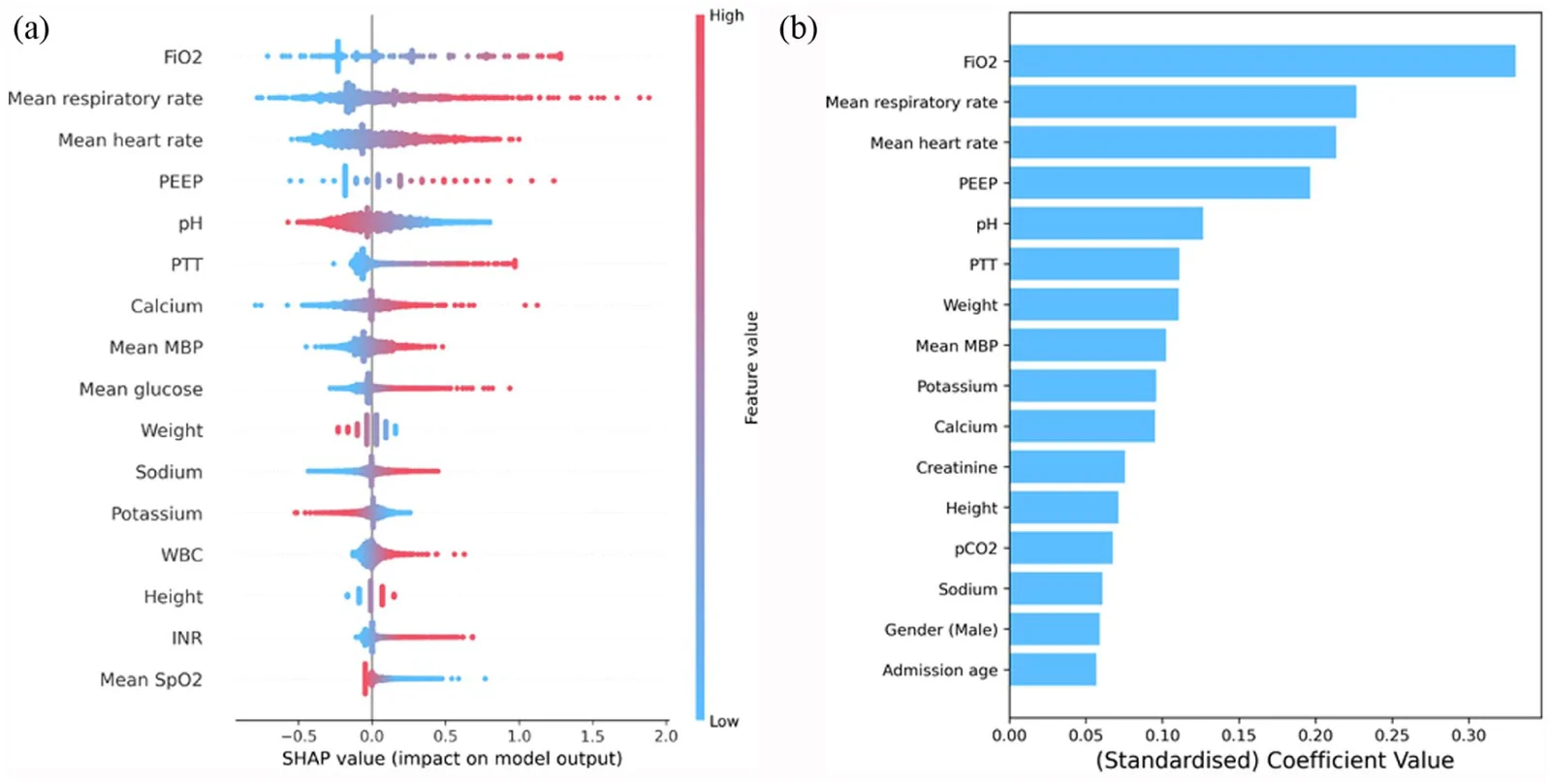
An overview of the most relevant features for the prediction of prolonged IMV in the full logistic regression model, based on: (a) SHAP attribution values and (b) standardised coefficients.

To derive a simplified and interpretable score, we examined feature importance using SHAP attribution values and standardised logistic regression coefficients (Figure 2). The most predictive subset of features comprised FiO_2_, PEEP, mean respiratory rate, mean heart rate, and the last measured pH. A reduced logistic regression model including only these variables retained good performance, with an AUROC of 0.749 (0.748–0.750).

**Figure 3.**
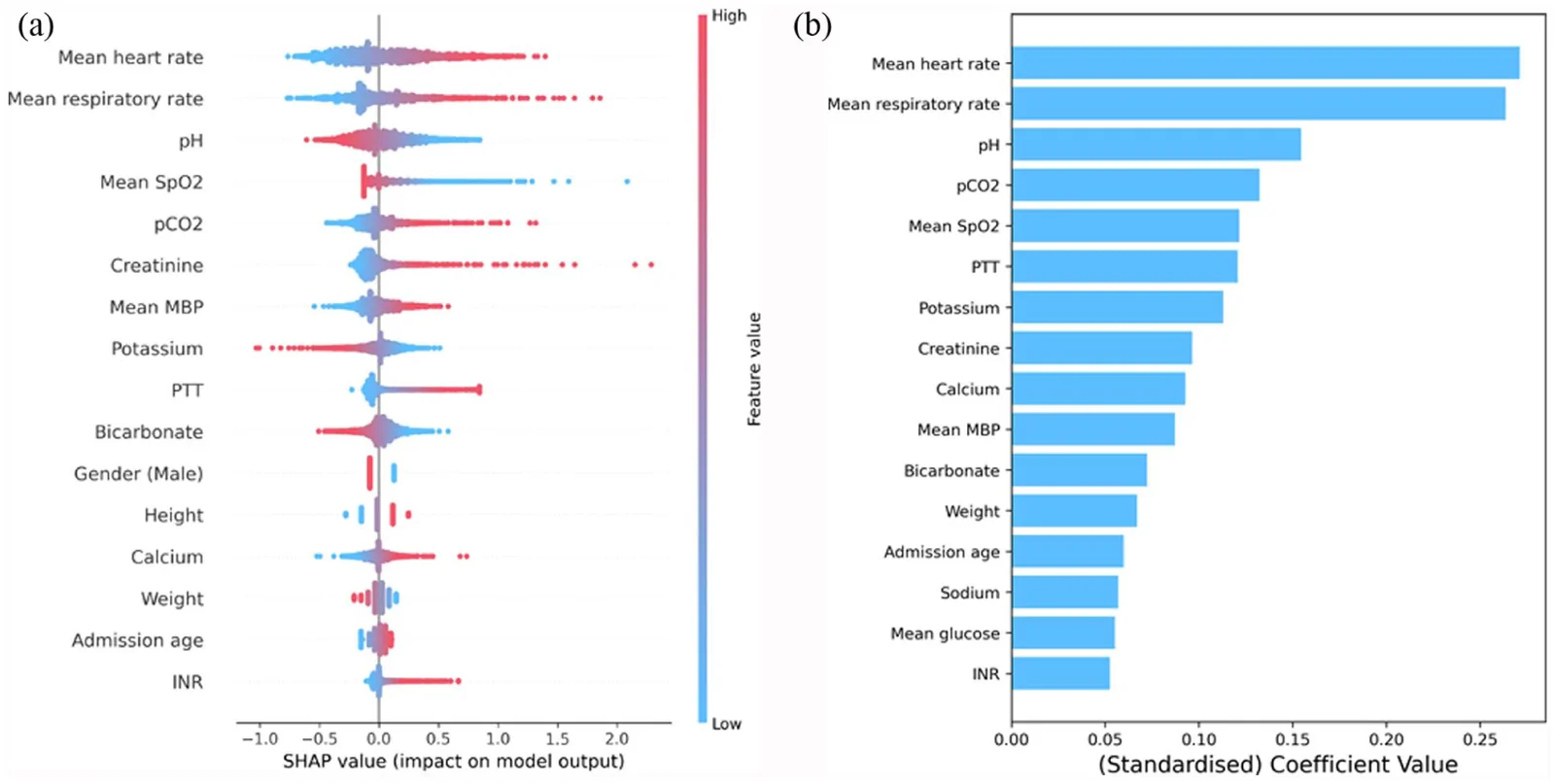
An overview of the most relevant features, excluding MV settings, for the prediction of prolonged IMV in the full logistic regression model, based on: (a) SHAP attribution values and (b) standardised coefficients.

Because ventilator settings (FiO_2_ and PEEP) may not be available at the time of intubation, we also derived an alternative feature set excluding these variables. Substituting FiO_2_ and PEEP with the mean SpO_2_ and last observed PaCO_2_ minimised performance loss (Figure 3). Using this subset, the model achieved an AUROC of 0.722 (0.720–0.723), representing a reduction of 0.027 points compared with the model including ventilator settings.

**Figure 4.**
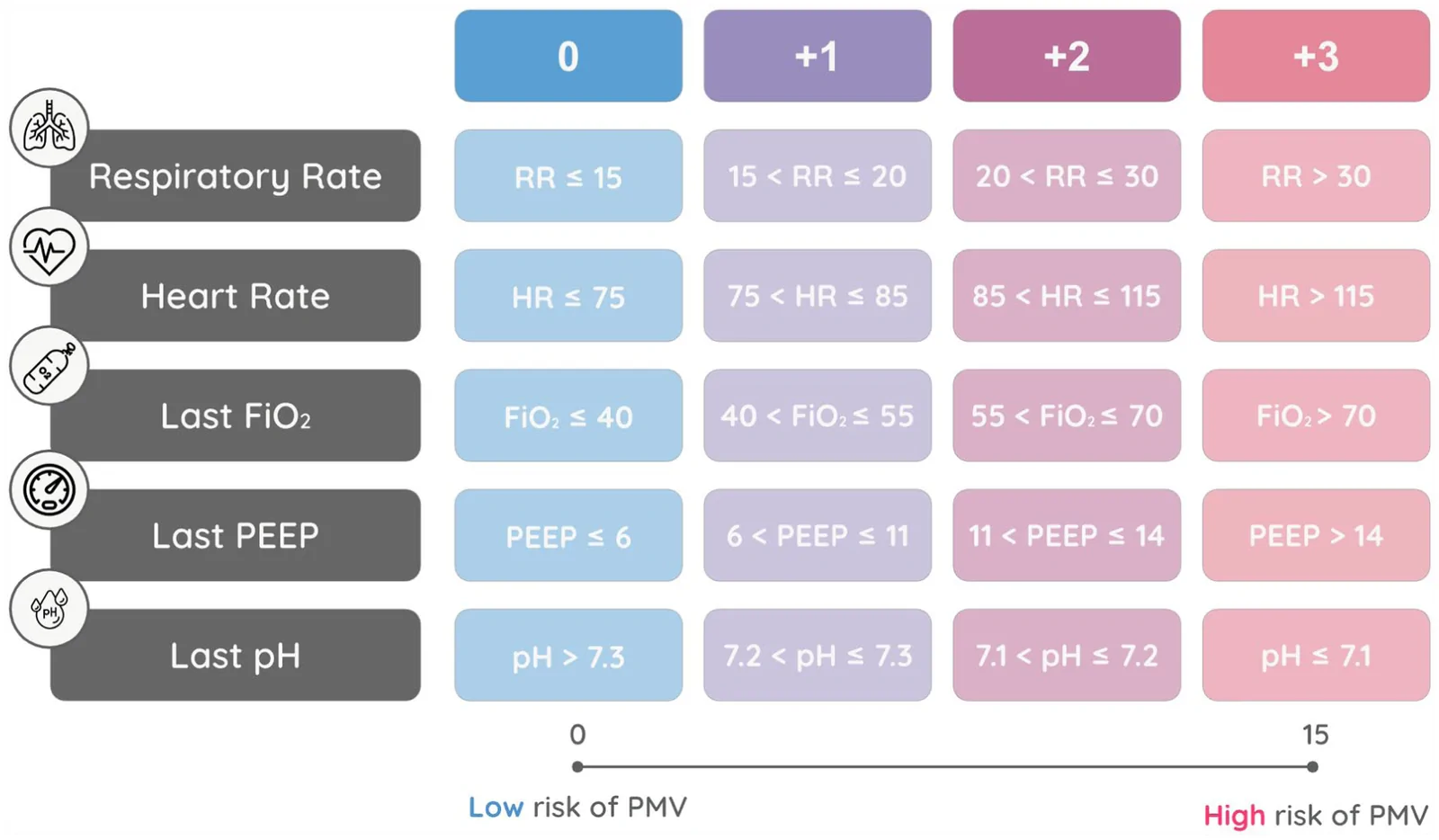
Visualisation of the ventilator-inclusive Length of Ventilation (LoVe) index score for predicting prolonged mechanical ventilation.

**Figure 5.**
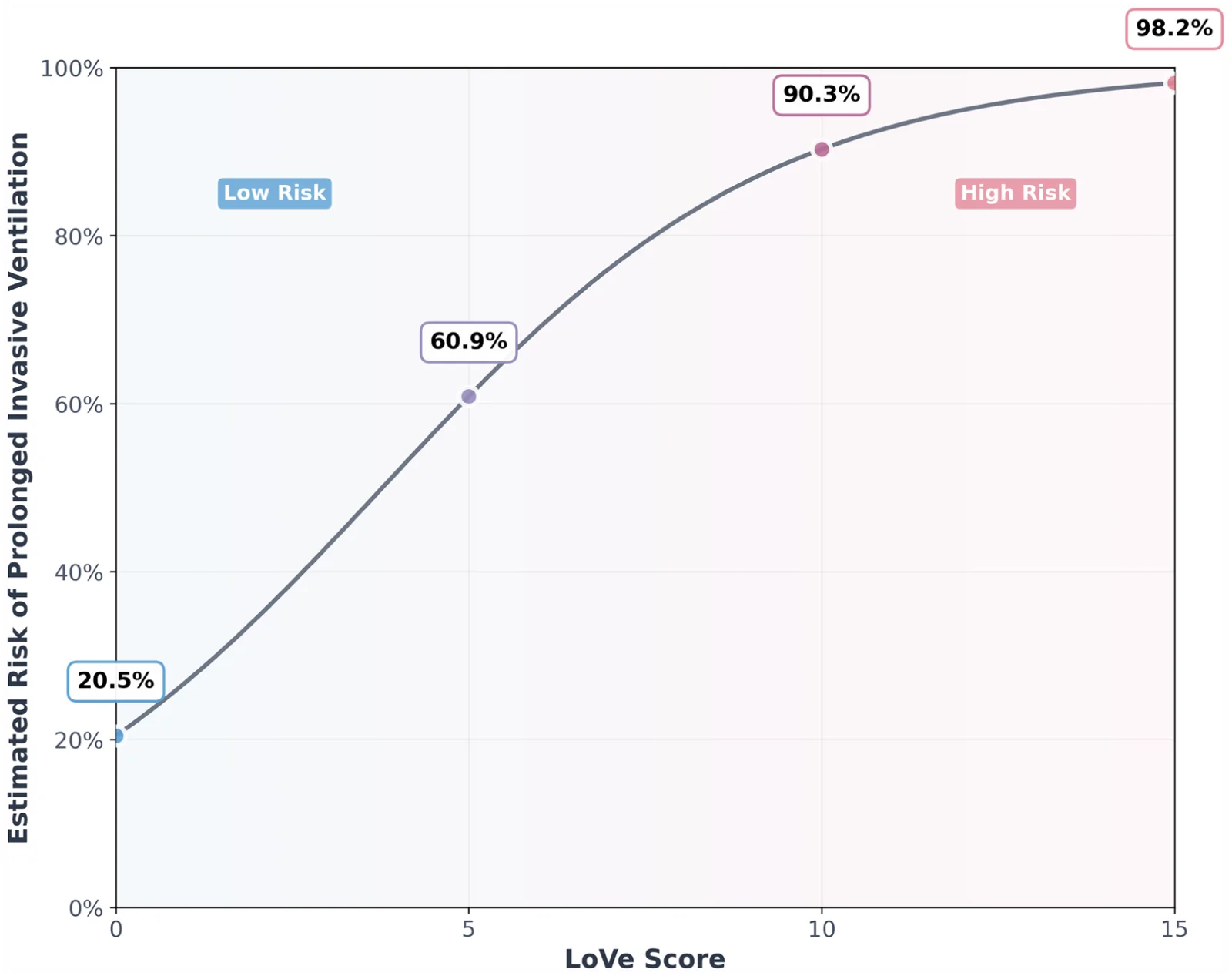
Relationship between the LoVe index score and estimated risk of prolonged invasive mechanical ventilation.

Guided by the feature selection above, we translated coefficients into a lightweight, point-based *Length of invasive mechanical Ventilation (LoVe)* index. Each predictor was discretised into clinically interpretable ranges based on an empirical assessment of the data distribution, and integer points (0–3) were assigned to each bin. A more elaborate description of the index score rubric derivation process can be found in Appendix A2. The resulting ventilator-inclusive scoring rubric displayed in Figure 4 provides a total score between 0 and 15 points, with higher scores indicating increasing risk of prolonged IMV. This index achieved an AUROC score of 0.750 (0.726–0.773), sensitivity of 0.614 (0.584–0.646), and specificity of 0.769 (0.738–0.799). Within the cardiac surgery cohort, the ventilator-inclusive LoVe score achieved an AUROC of 0.824 (0.772–0.870), sensitivity of 0.695 (0.600–0.788), and specificity of 0.802 (0.779–0.821). To illustrate the relationship between the cumulative LoVe score and estimated risk, Figure 5 displays the calibration curve mapping the index score value to the corresponding probability of prolonged mechanical ventilation.

**Figure 6.**
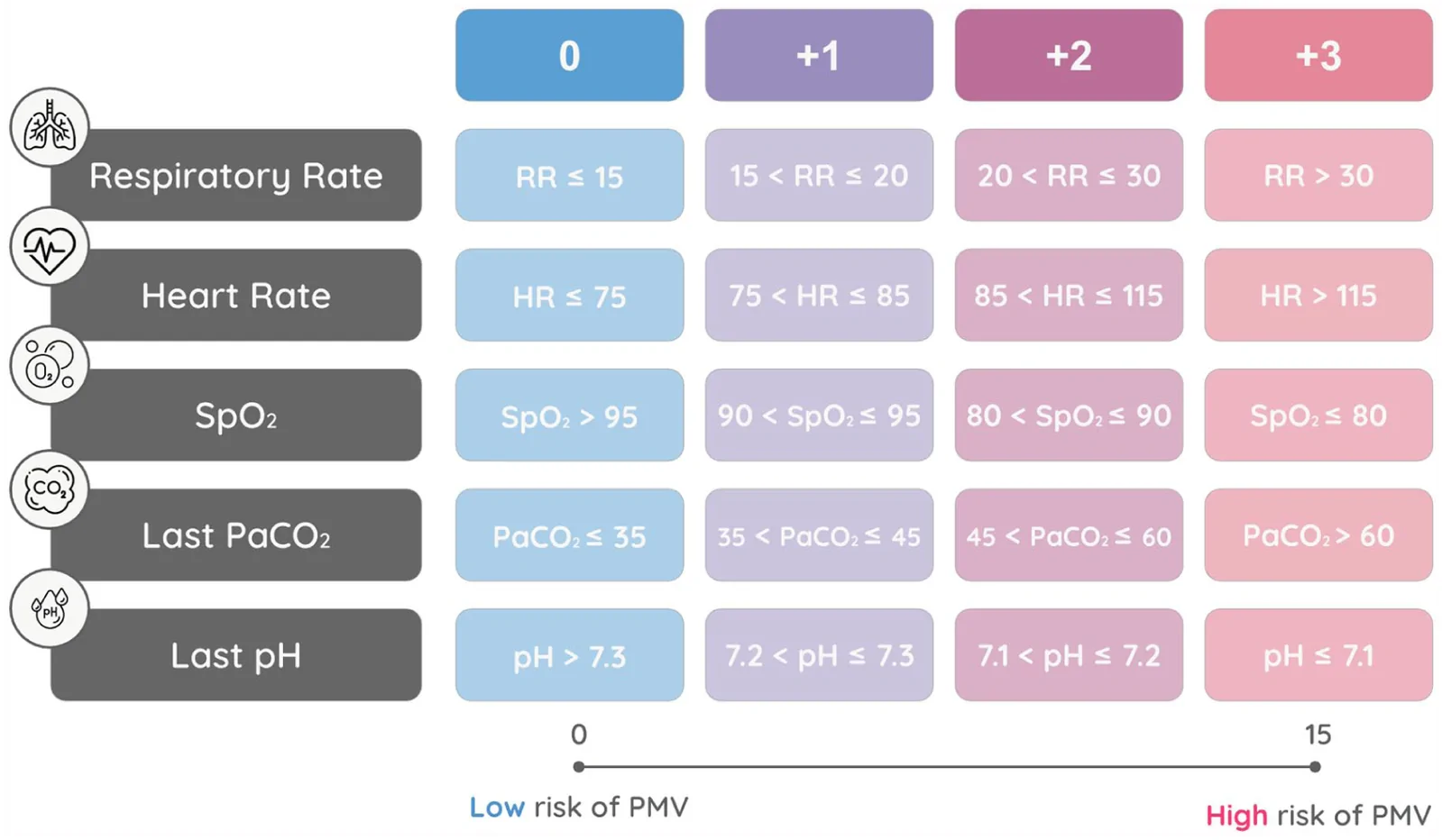
Visualisation of the ventilator-agnostic Length of Ventilation (LoVe) index score for predicting prolonged mechanical ventilation.

In parallel, we derived a ventilator-agnostic version of the LoVe index, based on SpO_2_ and PaCO_2_ instead of FiO_2_ and PEEP. This score retained the same 0–15 point framework (Figure 6) and achieved an AUROC of 0.718 (0.695–0.742), sensitivity of 0.498 (0.387–0.608), and specificity of 0.814 (0.737–0.888). This represents a reduction in AUROC of 0.032 points with respect to the ventilator-inclusive LoVe score.

In summary, both LoVe index variants retained comparable predictive performance, with modest differences between the ventilator-inclusive and ventilator-agnostic versions.

## The effect of timeframe

Having established the derivation and baseline performance of both LoVe index variants, we next evaluated predictive performance across different peri-intubation timeframes. In the non–cardiac surgery cohort, the ventilator-inclusive LoVe score achieved an AUROC of 0.756 (0.733–0.779), sensitivity of 0.606 (0.575–0.638), and specificity of 0.794 (0.764–0.824) when using the short-term window (0–1 h post-intubation). Performance was slightly lower for the long-term window (0–6 h), with an AUROC of 0.710 (0.684–0.734), sensitivity of 0.653 (0.622–0.683), and specificity of 0.653 (0.616–0.692). In the cardiac surgery cohort, the long-term window yielded an AUROC of 0.784 (0.731–0.834), sensitivity of 0.684 (0.590–0.781), and specificity of 0.774 (0.742–0.804).

## External validation

In order to assess the generalisability of the LoVe index, we validated both the ventilator-inclusive and ventilator-agnostic variants in the MIMIC-IV cohort, stratified by cardiac and non-cardiac surgery patients. In the non-cardiac surgery population, the ventilator-inclusive index achieved an AUROC of 0.678 (0.671–0.685), sensitivity of 0.465 (0.384–0.539), and specificity of 0.786 (0.730–0.846). The ventilator-agnostic version showed slightly lower performance, with an AUROC of 0.649 (0.641–0.657), sensitivity of 0.367 (0.276–0.459), and specificity of 0.831(0.768–0.892). In the cardiac surgery subgroup, we observed AUROC values of 0.708 (0.687–0.728) and 0.714 (0.695–0.733), a sensitivity of 0.462 (0.349–0.575) and 0.644 (0.535–0.754), and a specificity of 0.845 (0.783–0.906) and 0.677 (0.576–0.774).

## Decision Curve Analysis (DCA)

While metrics such as AUROC provide a valuable quantification of discriminatory performance and reliability, they do not directly indicate whether the use of a model would improve clinical decision-making. Indeed, the optimal trade-off between sensitivity and specificity or whether a predictive tool has added benefit at the bedside may vary across institutions, physicians or even circumstances. Decision curve analysis (DCA) addresses this consideration by estimating the net benefit of applying the LoVe index score to guide intervention strategy across a range of threshold probabilities, compared with default strategies of treating all or no patients. Although DCA results are agnostic to the specific intervention, they can be interpreted in light of potential bedside applications. For example, if the index were used to guide the initiation of active humidification at the time of intubation, the default strategies would correspond to providing this intervention to all or none of the patients. This analysis provides insight into the potential clinical utility of the index beyond statistical performance.

**Figure 7.**
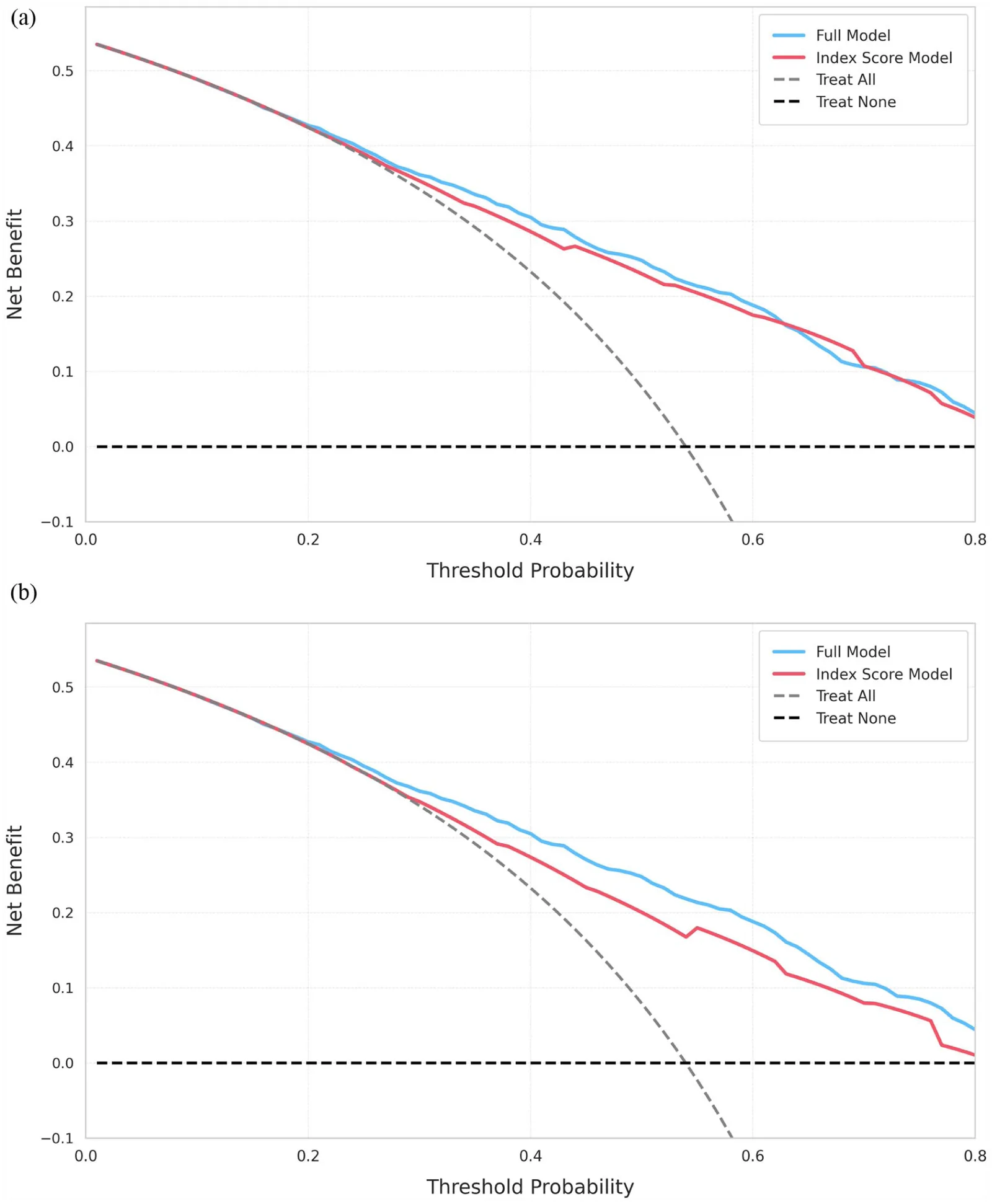
Decision curve analysis (DCA) for: (a) the ventilator-inclusive LoVe index and (b) the ventilator-agnostic LoVe index in the AmsterdamUMCdb non-cardiac surgery cohort. Net benefit (*y*-axis) expresses the value of using the index to guide treatment as the number of correctly identified prolonged IMV patients per patient assessed, after accounting for false positives. The threshold probability (*x*-axis) is the risk level at which a clinician would decide to act. The index offers clinical value whenever its curve lies above the “treat all” and “treat none” reference strategies, across the full range of relevant threshold probabilities.

**Figure 8.**
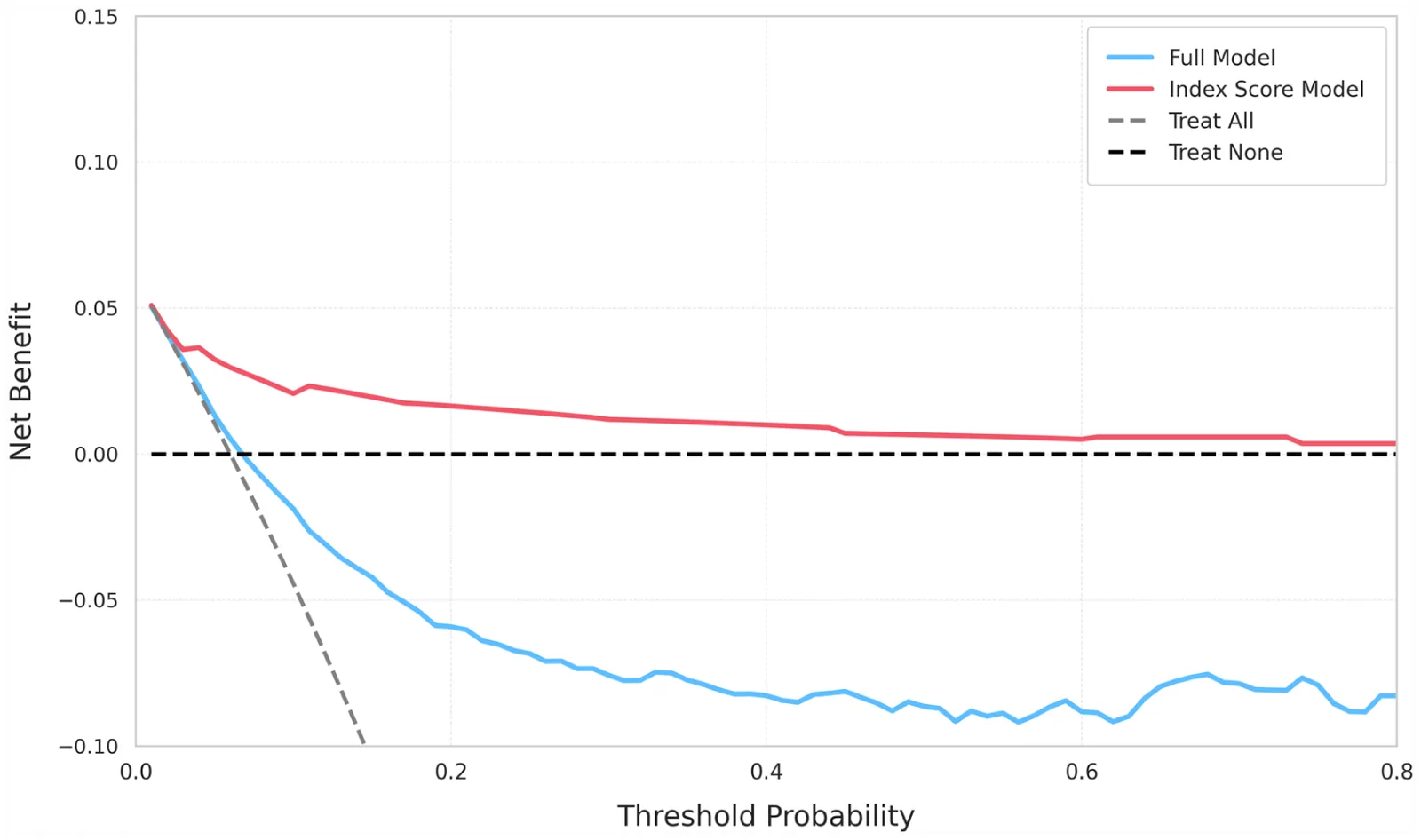
Decision curve analysis (DCA) for the ventilator-inclusive LoVe index in the AmsterdamUMCdb cardiac surgery cohort.

The DCA visualisations provided in Figure 7 reveal that both the ventilator-inclusive and ventilator-agnostic LoVe indices consistently provided equal or higher net benefit in the non-cardiac surgery cohort compared to the default strategies, across the entire evaluated range of risk threshold preferences. A similar pattern was observed in the cardiac surgery subgroup (Figure 8), although the overall magnitude of net benefit was smaller.

## Discussion

In this study, we propose a novel Length of Ventilation (LoVe) index, a simple and interpretable score to predict prolonged mechanical ventilation using peri-intubation data. Our findings suggest that both the ventilator-inclusive and ventilator-agnostic variants of the LoVe score provide solid predictive performance, with AUROC values of 0.75 and 0.72 for the non-cardiac surgery cohort, respectively. In the cardiac surgery population, the ventilator-inclusive LoVe score achieved an AUROC of 0.82. External validation in the MIMIC-IV cohort confirmed the generalisability of the score, with AUROCs of 0.71 and 0.68 for the ventilator-inclusive index in the cardiac and non-cardiac surgery cohorts, and 0.71 and 0.65 for the ventilator-agnostic version. Importantly, predictive performance remained stable across different peri-intubation time windows, supporting the practical flexibility of the tool.

Beyond discrimination, decision curve analysis demonstrated that both LoVe index variants provided consistent net benefit compared with default “treat-all” or “treat-none” strategies, in both non-cardiac and cardiac surgery patients. These findings highlight not only the robustness of the score across diverse patient populations and timeframes, but also its potential clinical utility in supporting early decision-making at the time of intubation.

Several prior studies have attempted to predict the duration of invasive mechanical ventilation, but our findings extend this work in important ways. Models that estimate ventilation duration in hours or days have demonstrated feasibility,^15–17^ yet their outputs are less directly aligned with bedside decision-making. As important clinical considerations, such as humidification allocation, are guided by dichotomous time horizons, we target this binary classification perspective directly. Methods that rely on data collected 48–72 h after initiation of ventilation or after the onset of ARDS have demonstrated strong performance.^16,17^ Unfortunately, the delayed prediction limits the applicability for guiding early interventions, as the opportunity to influence key decisions may have passed by the time the predictions become available. Similarly, models that define prolonged mechanical ventilation as support beyond 7 or 14 days provide useful longer-term prognostic information, but do not address management questions arising during or immediately after intubation. In contrast, the LoVe index is explicitly designed for use within the peri-intubation time window, when uncertainty about the likely course of ventilation is greatest and when clinicians are expected to make key decisions related to resource allocation and communication with families.

In addition to these more general approaches, prediction scores have also been developed specifically for cardiac surgery patients. The ANZ-PreVent score, for example, was derived from the Australian and New Zealand Society of Cardiac and Thoracic Surgeons (ANZSCTS) database^25^ to identify patients at risk of ventilation for more than 24 h, achieving a c-index of 0.84 in its derivation cohort.^13^ Its predecessor, the Modified Hessels score, reached a c-index of 0.78.^14^ While both scores demonstrated good discrimination within their intended setting, they rely on perioperative and surgical variables such as emergency surgery, intra-aortic balloon pump use, or cardiogenic shock. These predictors are relevant for elective postoperative care but are not necessarily relevant or even routinely available at the time of intubation in the broader ICU population. By contrast, the LoVe index is applicable across a heterogeneous group of critically ill patients and requires only routinely measured peri-intubation data, which supports broader generalisability and bedside use.

The LoVe index offers several potential applications in routine ICU practice. By providing an early, interpretable estimate of whether a patient is likely to remain ventilated beyond 48 h, the score could support decisions that hinge on expected ventilation duration. One relevant example is the choice between active or passive humidification strategy, where current guidelines and clinical practice rely on anticipated ventilation duration or delayed treatment. Similarly, the index could aid in identifying patients for whom early tracheostomy planning may be appropriate, or in informing families about the likely trajectory of care. Beyond individual patient management, consistent use of a simple but reliable index may help standardise practice across institutions, reduce variability in trial enrolment criteria, and facilitate more efficient allocation of ICU resources. As such, the intended use of the score is not to replace clinical judgement, but rather to provide an easy-to-use, transparent tool that complements the expertise of physicians.

We believe this study has several important strengths. First, it leverages two large, high-quality, publicly available ICU databases, enabling transparent development and external validation across distinct healthcare systems. Second, by limiting the model to five routinely measured variables and discretising them into an additive point-based score, we propose a tool that is simple, interpretable, and readily usable at the bedside. In fact, the feature composition in the model would most likely allow for automated calculation if the appropriate technical infrastructure is available. Third, performance was consistently evaluated across relevant subgroups, including cardiac and non-cardiac surgery patients, and across different peri-intubation timeframes, which supports the robustness and flexibility of the index. Finally, we incorporated a decision curve analysis to demonstrate potential clinical utility beyond statistical performance.

### Limitations

This study also has several limitations. First, the discriminatory performance of the LoVe index in terms of AUROC remains modest, presumably due to the deliberate design choices prioritising simplicity and interpretability. Although some other prediction models achieve comparable or marginally better performance, we emphasise that the value of the LoVe index lies in its accessibility and clinical alignment, rather than in marginal AUROC gains. Nonetheless, substituting the additive structure of the index with a formulation that captures interactions or non-linear relationships between predictors could potentially yield performance gains, and would represent a natural direction for a more complex successor model. Second, our model incorporates variables that are likely not themselves causal determinants of prolonged mechanical ventilation, such as ventilator settings like FiO_2_ and PEEP, which introduces the risk of misinterpretation. The observed settings reflect a clinical assessment of the patient and their inclusion should not be interpreted as suggesting that altering a ventilator parameter would change the patient’s risk of prolonged ventilation. This deliberate use of confounded associations is consistent with our aim to capture actionable prognostic information rather than mechanistic causality. Third, the model was restricted to a limited set of routinely available variables, and some potentially informative predictors could not be incorporated, such as admission diagnosis, a measure of frailty, or the duration of non-invasive ventilation preceding intubation. In addition, we explored the inclusion of pulmonary compliance as a feature but were restricted to a suboptimal estimate of dynamic compliance, due to the retrospective nature of the data (Appendix A1). As a result, its potential predictive contribution could not be reliably assessed and should be examined in future prospective studies. Fourth, for patients admitted to the ICU with a pre-existing tracheostomy, the true onset of invasive ventilation precedes ICU admission and therefore cannot be reconstructed from the available data, introducing a source of bias inherent to retrospective ICU databases. For these patients, the ventilation episode is anchored to the first documented ventilation event and use of the score should accordingly be interpreted as a reassessment of expected ventilation duration at ICU entry, rather than a prediction at the start of ventilation. Finally, while the LoVe index was developed and externally validated using large observational cohorts, its clinical value remains to be established in prospective validation studies.

## Conclusions

In summary, we developed and validated the Length of Ventilation (LoVe) index, a simple, interpretable bedside score to estimate the risk of ventilation duration exceeding 48 h, using peri-intubation data. The LoVe index demonstrated consistent solid performance across patient subgroups and timeframes, and a decision curve analysis indicated tangible potential for clinical utility. By translating routine clinical variables into an easy-to-use additive score, the LoVe index provides a practical tool to support early management decisions at the time of intubation. Future prospective validation is warranted to determine its impact on clinical practice and patient outcomes.

## Figures and Tables

**Figure A1.**
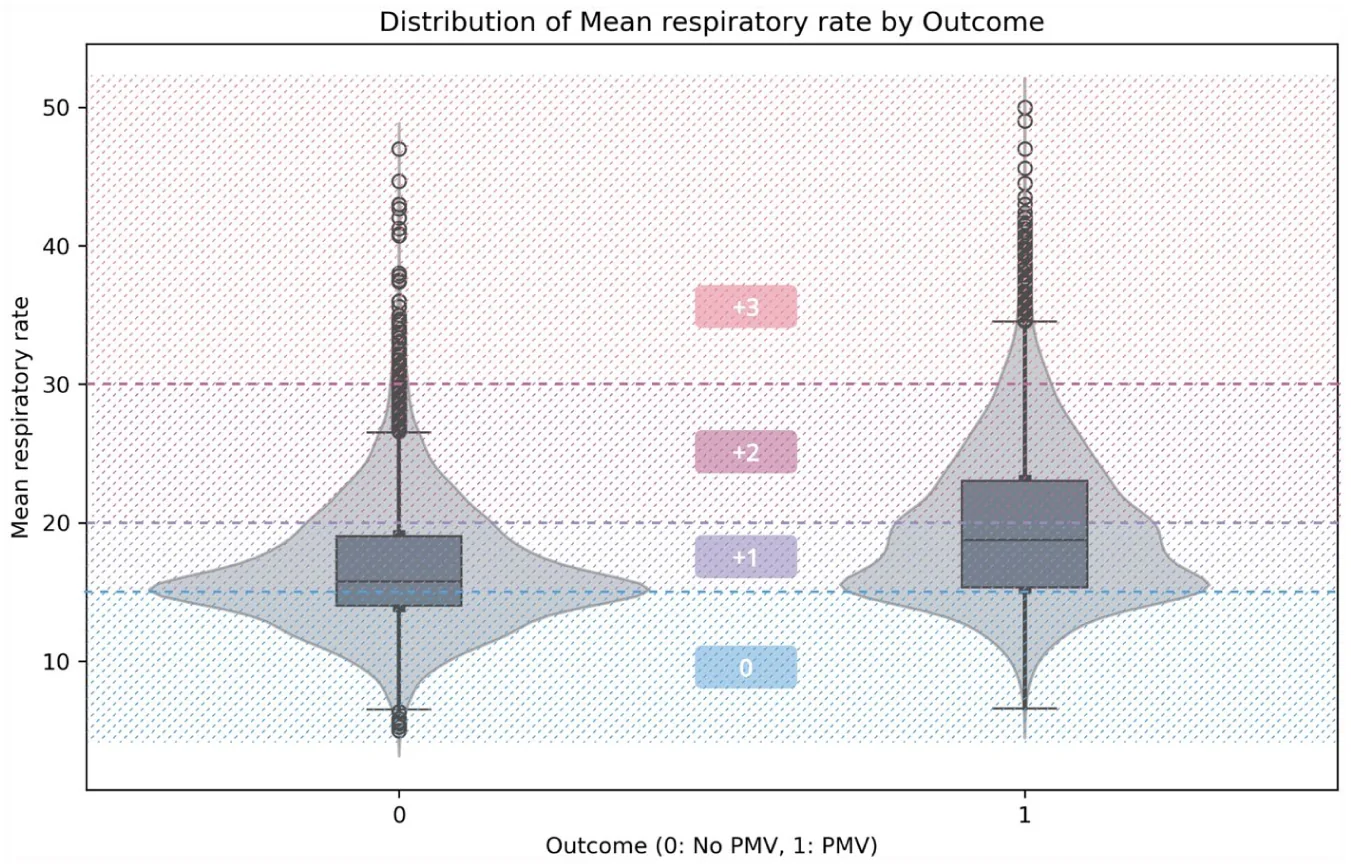
A demonstration of feature binning for the mean respiratory rate variable within the LoVe index score rubric. Each resulting range was then assigned an integer score between 0 and 3, with the direction of the corresponding regression coefficient determining whether higher or lower values increased the score, and the bin boundaries placed at the points where the prolonged and non-prolonged IMV distributions began to diverge most clearly. Where it did not compromise this separation, boundaries were rounded to clinically familiar values to ease bedside use.

## Appendix

### A1 – Estimating dynamic compliance for use as a predictor

Given its clinical relevance for assessing the respiratory state of a patient, we believe compliance to be a potentially valuable feature for the prediction of prolonged mechanical ventilation. Unfortunately, the retrospective nature of the data limited the accuracy of attainable compliance estimates. As the group of patients who received an inspiratory hold manoeuvre was both small in number and likely non-randomly selected, a reliable measurement of static compliance was not available. Instead, we used two equations to derive an estimate for dynamic compliance, depending on data availability. If a measurement for plateau pressure was available, we used the following estimate for dynamic compliance:

$$C_{dyn}=\frac{V_{t}}{P_{plat}-PEEP}$$

Where $V_{t}$ refers to the tidal volume, PEEP denotes the Positive End-Expiratory Pressure, and $P_{plat}$ is the plateau pressure measurement provided by the ventilator. Note that these plateau pressure measurements differ from those obtained during a formal inspiratory hold manoeuvre.

When plateau pressure was not available, we used the following equation instead:

$$C_{dyn}=\frac{V_{t}}{PIP-PEEP}$$

Where PIP refers to the measured Peak Inspiratory Pressure.

In our experiments, inclusion of our derived approximation of dynamic compliance did not meaningfully improve predictive performance. While the feature did yield predictive value, it primarily seemed to provide a solid substitute for PEEP. Given the added complexity and limited incremental benefit, dynamic compliance was excluded from the final feature set.

Nonetheless, pulmonary compliance remains a physiologically important marker and may provide additional predictive value beyond the features included in the current model. Prospective validation of the LoVe index should therefore consider incorporating reliable static compliance measurements to assess their added predictive value.

### A2 – Derivation of the LoVe index ranges

After identifying the subset of most predictive features, we discretised the continuous predictors into clinically interpretable ranges. Cut-off values were chosen based on visual inspection of the distributions in patients with and without prolonged IMV, supported by descriptive statistics from the AmsterdamUMCdb training dataset. Figure A1 illustrates this process for the mean respiratory rate variable, showing how discrete bins were defined for use in the LoVe index rubric.
